# How will the public health committees develop after COVID-19 pandemic in China? Exploration from mixed methods study in Pingshan District, Shenzhen

**DOI:** 10.3389/fpubh.2024.1307771

**Published:** 2024-01-17

**Authors:** Xu Shao, Xiangling Wu

**Affiliations:** ^1^School of Political Science and Public Administration, Wuhan University, Wuhan, China; ^2^School of Electronic Information and Artificial Intelligence, Leshan Normal University, Sichuan, China

**Keywords:** public health committees, community, Pingshan District, autonomization, administration

## Abstract

**Introduction:**

The Public Health Committee has a long-standing presence in the Chinese Constitution. During the pandemic, it served as a grassroots self-governance organization and made significant contributions to China's community epidemic prevention and control system. Currently, 24 provinces in China have promoted the establishment of community public health committees.

**Methods:**

To gather data, we conducted semi-structured interviews (*n* = 48) with the heads of superior departments of public health committees, the heads of public health committees, and the heads of community health centers, exploring aspects such as organizational structure, job responsibilities, and job security. In parallel, we administered a capacity-building survey to a sample of 23 community residents (*n* = 1,986) and performed regression analysis. Finally, we examined the impact of gender, age, and education level on the development of public health committees.

**Results:**

Our study reveals that the development of public health committees displays features of administration across various dimensions, including personnel appointment, top-level design, medical professionalism, funding path dependence, and data path dependence. However, the decision-making function of the organization does not exhibit a significant impact.

**Discussion:**

The construction of the Public Health Committee should demonstrate an “autonomization-administration” pendulum effect. Currently, due to the absence of decision-making functions within the Public Health Committee, autonomous organizations are exhibiting characteristics of administration. To prevent excessive autonomization or administrative nature in the development of committees, public health policies are continuously being refined based on the unique characteristics of public health committee construction. During exceptional circumstances or the initial stages of development, the establishment of public health committees should be primarily guided by administrative principles, utilizing political momentum to drive their progress. In contrast, during routine establishment phases or later stages of development, the establishment of public health committees should be primarily led by autonomization, restoring their capacity for self-decision making. It is essential to fully leverage the role of grassroots self-governance organizations, relying on the community to engage in self-management, self-education, and self-service within public health committees.

## 1 Introduction

The public health committee first appeared in the Constitution of the People's Republic of China in 1982. Article 111, paragraph 2 stipulates that village committees should establish public health committees (1). However, the specific composition of the members, responsibilities, operating mechanism, and institutional guarantees have not been specified. In the Organic Law on the Villagers Committee of the People's Republic of China (for Trial Implementation) adopted in 1987, there was a provision that was inconsistent with the Constitution that the establishment of a village health committee was based on the needs of the villagers committee. Villages with a small population did not have to establish one, and the villagers committee could share public health work. Subsequently, in the revised Organic Law on the Villagers Committee of the People's Republic of China in 1998 and 2018, only the name of the Public Health Committee was changed. The legislative thinking of the central government has profoundly influenced local legislation (2). In the laws and regulations formulated by local governments, most provinces only mention the establishment of public health committees, and some provinces do not even mention any content related to public health committees. The lack of attention to public health committees has also contributed to the difficulty in playing a role in grassroots public health governance over the past three decades of development.

It wasn't until the Healthy China policy was promoted to a national strategy that some local governments started to explore the establishment of public health committees (3). In 2018, the Beijing Municipal Health and Family Planning Commission first came up with a policy which stated that Beijing had basically set up a public health committee system by 2020. They had to specify the personnel composition, institutional responsibilities, funding usage, and other functions of the public health committee. It included patriotic health work, basic public health services, epidemic prevention and control, health education, birth planning, and other responsibilities. As of July 2021, Beijing became the first place in China to have full coverage of grassroots public health committees (4).

During the pandemic, members of the public health committee not only participated in controlling the sources of infection and cutting off the channels of transmission, including epidemic monitoring, taking measures to help returnees from infected regions in order to prevent any possible spread of the coronavirus and assisting with epidemic management. They also leveraged and integrated grassroots health resources to maximize the contributions of public health service centers, community service centers, village health clinics, property managers, and volunteers to pool their efforts in epidemic prevention and control (5). There are two fronts in the fight against the epidemic. One is the hospital setting for saving lives and curing the sick, and the other is the community setting for containing the spread of the virus. The key to unflagging efforts in epidemic prevention and control lies squarely in the community (6). As a grassroots self-governing organization in the community, the public health committee's capabilities in epidemic emergency response, mobilization and coordination of resources have been increasingly recognized by the public during the pandemic (7).

The central government and local authorities have reviewed the role and establishment of public health committee. Therefore, in 2021, the Ministry of Civil Affairs, the National Health Commission, the State Administration of Traditional Chinese Medicine, and the China Center for Disease Control and Prevention jointly issued the policy about Guiding Opinions on Strengthening the Construction of Public Health Committee in Village (Residents') Committee, which aims to achieve full coverage of public health committee mechanisms, enhance overall capacity, and effectively utilize their roles. It is expected to establish a grassroots public health management mechanism with dynamic convergence between normal management and emergency management, making positive contributions to implementing the strategy of Healthy China, promoting whole process people's democracy, and modernizing grassroots governance (8). The key focus of local government public health committee construction also centers around how to effectively respond to the COVID-19 epidemic. The specific functions of committee mainly include two parts. Firstly, it is essential to collaborate with all stakeholders and coordinate efforts to effectively address key pandemic prevention and control efforts. Secondly, it is crucial to organize and mobilize efforts to effectively deliver essential public health services within the jurisdiction, collaborate on vaccination programs, and gather and report vital health information including the epidemic situation.

On May 5th, 2023, the World Health Organization declared that the COVID-19 pandemic is no longer a public health emergency of international concern (9). This declaration marked a significant step toward bringing the pandemic to an end. In the post-pandemic era, not only the central government but also local governments in various provinces are focusing on how to establish the public health committee that can play an equally crucial role as they did during the pandemic. In our research, members of public health committee have expressed the need for more specific guidelines to direct their work in the post-pandemic era. “*It is everyone's hope to have a more normalized mechanism to help guide our efforts.” (L-HY-20235152)*

Guangdong Province is one of the earliest provinces in China to pay attention to the construction of public health committee. At the end of 2021, Guangdong Province required all villages in the province to establish public health committees, enhancing villagers' public health awareness and satisfaction with public health services. So Pingshan District in Shenzhen completed the establishment of 23 community public health committees in June 2021.

Guangdong Province is one of the earliest provinces in China to pay attention to the construction of public health committee. At the end of 2021, Guangdong Province required all villages in the province to establish public health committees, enhancing villagers' public health awareness and satisfaction with public health services. Pingshan District in Shenzhen completed the establishment of 23 community public health committees in June 2021. During the COVID-19 pandemic, Pingshan District had explored a “three-in-one” working model for public health committee that community staff, community medical staff and community police played key roles in the investigation of suspected cases, home quarantine health management, nucleic acid sampling, and vaccination. Additionally, public health committee had partnered with biopharmaceutical and healthcare enterprises to formulate the policy of A Ten-item Action List for a Healthy Pingshan District, which focused on ten actions for promoting healthy communities such as health education, convenient medical diagnosis, traditional Chinese medicine inheritance, and emergency response training. Finally, public health committee had established health service stations that serve as the main platform for Pingshan District's public health committee to perform their duties and carry out various health activities. Overall, Pingshan District's public health committee construction is representative in terms of organizational structure, mechanism innovation, and resource coordination.

While public health committee has always been present in China's Constitution. The government only began to prioritize their development during the pandemic. So far, existing research has mainly focused on discussing the ideas for building public health committee in China without presenting specific actionable plans (10). At the practical level, strict top-down bureaucratic management is a significant institutional factor that shapes China's policy style (11). For the most part, public health committee construction in various provinces follows the central government's guidance, often without consideration for local circumstances, resulting in a one-size-fits-all approach. In fact, some provinces or cities haven't even started building public health committee.

**Figure 1:**
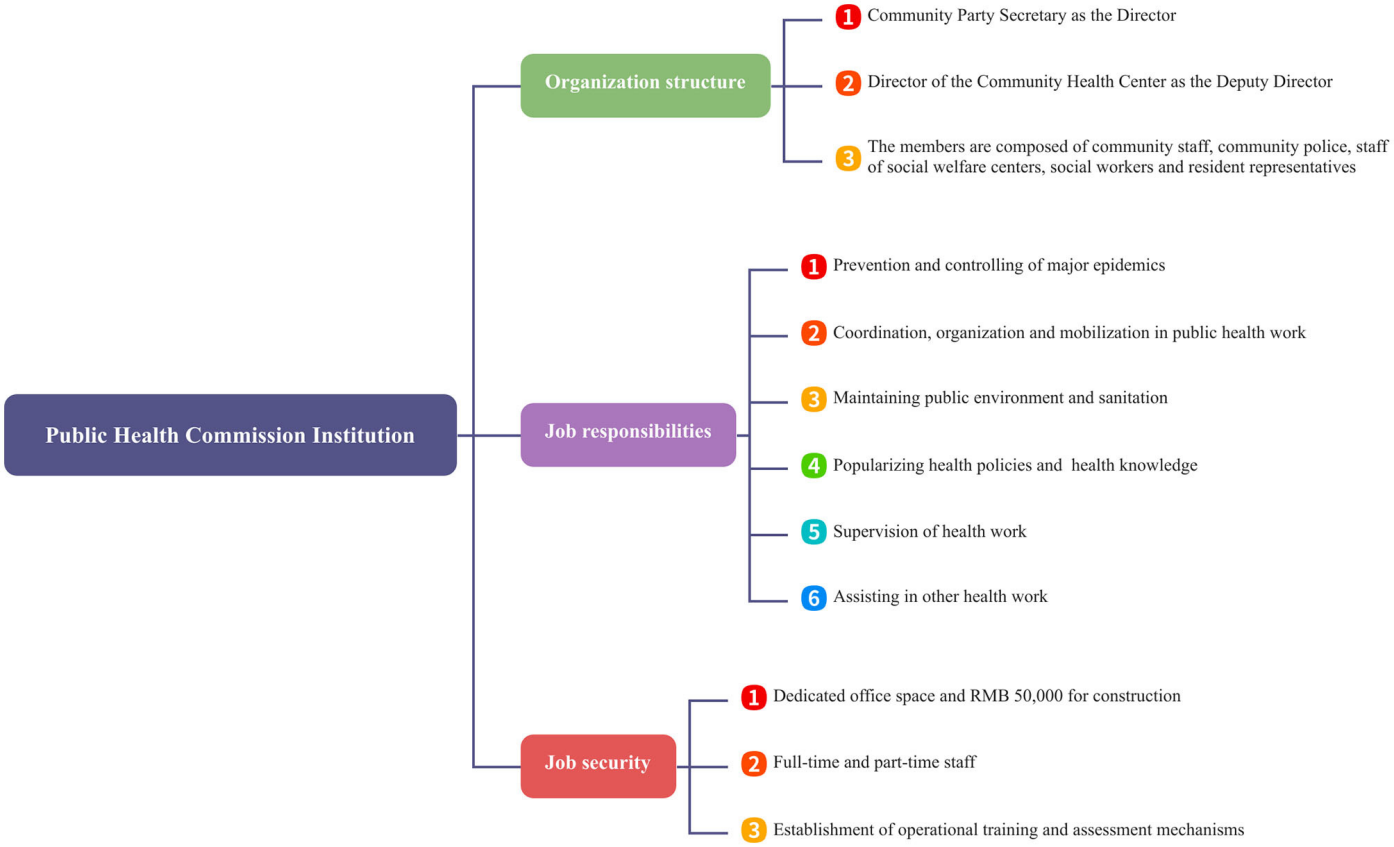
Construction of the specific content for the committees in Pingshan District.

Currently, public health committee only has a preliminary theoretical framework that needs to be further refined and combined with local circumstances for construction. This study fills the research gap by conducting a detailed investigation of the current situation of public health committee construction in 23 communities of Pingshan District, Shenzhen, exploring the characteristics of experience and the existing problems. Pingshan District government has issued policies for the construction of public health committee, specifying the organizational structure, job responsibilities, and work guarantee. The specific content is shown in Figure 1.

## 2 Method

### 2.1 Research design

This was a mixed-methods study, collecting qualitative and quantitative primary data in a sequential manner (12). This research focuses on optimizing the construction of public health committees from four perspectives: organizational structure, job responsibilities, job security, and capacity building. Guided by the Pingshan District Government's public health policies, we conducted extensive qualitative research on the dimensions of organizational structure, job responsibilities and job security. The study mainly involved semi-structured interviews with leaders of the Health Bureau of Pingshan District, street officials, staff members of the Public Health Committee, and community health center directors. We also conducted field observations and document analysis to triangulate the data collected through interviews.

Capacity building for public health committees mainly focuses on enhancing awareness of their functions, including executive, decision-making, coordination and communication, and organizational mobilization functions. Using the general functions of public health committee as explanatory variables and satisfaction with grassroots public health service capacity as the explained variable, this study aims to explore the influencing factors and existing issues of capacity building for public health committee through quantitative research.

Currently, primary public health services in Shenzhen are mainly provided by public health committee and community health service centers. As the grassroots self-governing organization, public health committee possesses the characteristics of self-management, self-education, self-service, and also has executive, decision-making, coordination and communication, and organizational mobilization functions in primary public health services (13). Shenzhen is one of the first cities to establish community health service centers in China, and after more than a decade of development, thousands of such centers have been established. In primary public health services, community health service centers play an important role in providing basic diagnosis and treatment services. Therefore, capacity building involves conducting a questionnaire survey among residents of all 23 communities in Pingshan District.

### 2.2 Basic information

Pingshan District is located in the northeast of Shenzhen, Guangdong Province, and was established in 2017 as a relatively young administrative region. It was also one of the first administrative regions in Shenzhen to promote the construction of public health committees. During the pandemic, Shenzhen government issued the “Notice on Promoting the Construction of Residents' Committee Public Health Committees,” requiring the establishment of public health committees to quickly play a role in the prevention and control of the COVID-19 epidemic in communities. By June 2021, 23 communities in Pingshan District had completed the establishment of community public health committees. The chairman of the public health committee is usually the community party committee secretary, and the vice chairman is usually the community health center director. The members include members of the epidemic prevention and control team (community staff, community police, and community health center staff), residents' representatives, members of the property service enterprise, etc. During the epidemic period, they worked together with Pingshan District community health service centers to coordinate and carry out epidemic prevention and control work.

### 2.3 Study participants

Before the research, all procedures were carried out in accordance with relevant regulations and rules. Interviewers were informed of the procedures and interview outline before the interview, and obtained informed consent from all participants. To protect the privacy of interviewees, no personal information was collected during the audio recording of interviews, and interview records were coded using anonymous identifiers to ensure participant privacy. The code uses the initials of the street or community, the abbreviation of the interviewer's last name, and the detailed time in month and date format. AM is coded as 1 and PM is coded as 2.In the questionnaire survey, the purpose of the survey was explained to the respondents, and the questionnaire introduction also provided a detailed explanation of the specific purpose of the survey. The questionnaire did not collect any personal privacy-related information, such as name, specific address, and ID number, etc.

In the in-depth interviews, specific information was gathered, as shown in Table 1, focusing on the organizational structure, job responsibilities, and job security related to public health committee construction. We interviewed two people in charge from Pingshan District Public Health Bureau, as well as the heads of public health committees and community health service centers from 23 communities, to gain a deep understanding of the implementation of policies related to public health committee construction. Regarding capacity building, 3,000 paper questionnaires were distributed with a 95% confidence level and a 5% margin of error based on the total number of target population in each community.

**Table 1:** Data collection.

**Dimensionality**	**Interview method**	**Participants**	**Number of interviews**
Organization structure	In-depth interviews	Policy makers	2
		Community secretary	23
		Director of community health service	23
Job responsibilities	In-depth interviews	Policy makers	2
		Community secretary	23
		Director of community health service	23
Job security	In-depth interviews	Policy makers	2
		Community secretary	23
		Director of community health service	23
Capacity building	Questionnaires	Local residents	2,000

## 3 Data collection

### 3.1 Quantitative

Trained interviewers collected the survey data using a structured questionnaire. With the assistance of the local health department, experienced community staff were identified to assist in data collection. The community interviewers were trained on the requirements, procedures, and guidelines for the paper questionnaire survey. The questionnaire aims to build the community's public health service capacity. This article takes satisfaction with public health service capacity building as the explained variable. The satisfaction rating scale is that “Very Satisfied” encoded as 5. “Satisfied” encoded as 4. “Average” encoded as 3. “Dissatisfied” encoded as 2. “Very Dissatisfied” encoded as 1.

The level of recognition for the public health committee is the explanatory variable in this study. This recognition is demonstrated through functional execution. Therefore, we have measured the level of recognition from the framework of general functions of mass self-governing organizations, divided into four dimensions: execution function, coordination and communication function, decision-making function, and organizational mobilization function (14). The execution function mainly reflects the problem of disconnect between community medical service supply and resident demand, reflecting the effectiveness of execution function. The higher the degree of disconnect between supply and demand, the poorer the effectiveness of execution function (15). The coordination and communication function mainly reflects the number of channels that residents can obtain communication. The more communication channels, the better the coordination and communication effect will be (16). The decision-making function mainly reflects that the public health committee can independently provide medical and health services to residents. The more medical and health services that can be provided, the better the decision-making function effect will be (17). The organizational mobilization function mainly reflects the number of channels residents can be organized and mobilized. The more organizational mobilization channels, the better the organizational mobilization effect will be (18). Before starting the formal questionnaire data collection, a pretest was conducted to modify problems and potential errors. Some concepts that were not familiar to local residents were adjusted to adapt to the research environment. Additionally, members of the public health committee conducted a pilot test on the questionnaire to validate basic variables through triangulation data collection to alleviate the influence of other factors.

Based on the above analysis, we propose the following hypothesis:

Hypothesis 1: The awareness of public health committee significantly positively impacts satisfaction with primary public health service capacity.

In particular:

Hypothesis 1a: The higher the level of execution function, the greater the improvement in satisfaction with primary public health service capacity.

Hypothesis 1b: The higher the level of coordination and communication, the greater the improvement in satisfaction with primary public health service capacity.

Hypothesis 1c: The higher the level of decision-making function, the greater the improvement in satisfaction with primary public health service capacity.

Hypothesis 1d: The higher the level of organizational mobilization, the greater the improvement in satisfaction with primary public health service capacity.

In order to account for potential bias introduced by excluding key variables, several additional control variables were incorporated into the analysis. These controls include age, gender and education level. Specifically, ages of residents are under 18 that are assigned a value of 1. The residents in age of are assigned a value of 2, and those over 61 are assigned a value of 3. Men are assigned a value of 2. Women are assigned a value of 1. And education levels range from 1 for high school or below, to 6 for doctoral degrees. A total of 2,146 questionnaires were collected, of which 1,968 were valid, resulting in a response rate of 91.7%. Data collection took place between May and June of 2023.

### 3.2 Qualitative

All interviews were carried out by professional members of the team. The interview with the person in charge of the Health Bureau (policy maker) was conducted at the Health Bureau's workplace in Pingshan District, lasting for an average of 2 to 3 h. The semi-structured interview centered on the basic situation, distinctive measures, obstacles, and future development trends of community public health committee construction. Interviews with other community public health committee heads and community health service center heads were conducted at the office premises of their respective districts, lasting for an average of 50 to 60 min. The interviews focused on guidance for community public health committee construction, implemented service projects, promotion of health and wellness knowledge, supervision channels, obstacles, future development trends, and satisfaction evaluation. The interviews were conducted between May and June 2023.

## 4 Analysis

In qualitative research, recordings of policymakers, heads of community public health committees, and heads of community rehabilitation centers are transcribed into text using software. The interviewers review their audio files to ensure accuracy. Using the analytical method of the subject framework, the interview data is analyzed (19). First, interviewers read through the interview transcripts and become familiar with the interview content. Then, they extract key ideas and key phrases from the interview outline and interview content. Classify them into three themes: organizational structure, job responsibilities, and job security. Finally, they interpret the results of each theme classification. Supportive citations from the interviews are included.

In quantitative analysis, we first conducted descriptive analysis of the dependent variable, independent variables, and control variables. Then, to examine the impact of public health committee cognitive ability on primary public health service capacity, we chose to use an ordered-probit model for analysis. This model assumes the existence of a latent variable (latent grassroots public health service capacity, GPHSC^*^) that can represent the dependent variable (primary public health service capacity, PHS) but cannot be directly measured. It requires the following selection conditions to be met: when μ_j − 1_ < y${\;}^{*}<\mu_{\text{j}}$, y = j (j = 1, 2, 3, 4, 5). Therefore, the regression equation for grassroots public health service capacity is:


$$\begin{array}{l} {\text{GPHSC}}_{\text{i}}^{*}=\beta_{0}+\beta_{1}CA_{i}+\beta_{2}C_{i}+\varepsilon_{i} \end{array}$$

In the equation, CA_i_ is an indicator that measures public health committee cognitive ability (including supply and demand, information channels, organizational mobilization ability, emergency response ability). And β1 represents the corresponding regression coefficient. C_i_ is an individual variable that affects grassroots public health service capacity. β_2_ represents the corresponding regression coefficient matrix, and ε_i_ symbolizes random error. Finally, we test the hypothesis in this research and discuss the potential endogeneity issue by introducing the street-level average cultural level.

## 5 Results

### 5.1 Qualitative findings

#### 5.1.1 Governance subject

##### 5.1.1.1 Composition and requirements of personnel

Although the public health committee belongs to grassroots mass organizations. According to the specific regulations on the Organic Law of the Villagers Committee of the People's Republic of China and the Organic Law of the Neighborhood Committee of the People's Republic of China, members of grassroots mass organizations are elected and self-governance, self-education, and self-service are practiced (20). In the construction of the Public Health Committee in Pingshan District, District Ministry of Organization, District Public Health Bureau and District Civil Affairs Bureau jointly issued regulations stipulating that the chairman of community public health committee should be appointed by secretary of party committee in the community, and the deputy chairman should be appointed by the community health service center director. The staff should be composed of community staff, community police, community health center staff, resident representatives, property service enterprises, etc. During the epidemic period, the establishment of the public health committee, like other community organizations, has an obvious administrative characteristic (5). Although later policies also require communities to hold residents committee meetings to discuss and vote on the appointment of public health committee chairmen, deputy chairmen, and members. In the post-epidemic era, some community public health committees have not implemented the policy according to the regulations. The organization members still only use the previous community staff. “*There are two teams in the community that one is the party committee and the other is the residents committee, which are legally required to be elected. Everyone knows that however the public health committee has not been elected.” (SH-C-5161)”During the epidemic period, the public health committee was quickly established to be responsible for epidemic prevention and control and nucleic acid testing. But there were no actual changes in personnel.” (WJJ-C-5151)*

The composition of public health committee members through appointment by the higher government rather than elections has resulted in issues such as insufficient staffing, low enthusiasm, high turnover, and lack of professionalism. “*Although the community public health committee has community secretaries, resident representatives, property management companies, business representatives, and community health center personnel. The main work is being done by only two people, including myself.” (KX-H-5172) “The public health committee does not have professional medical personnel and the sufficient staffing, and the salaries are not high, which all lead to a high turnover rate. In addition to the work of the public health committee, I am also responsible for the community women's federation and children's work, as well as daily routines.” (HP-S-5152)* Then, due to the presence of public and private community health centers in some neighborhoods, this alleviates the primary public health resource shortage problem (21). However, the policy does not provide specific details on the composition of the public health committee, particularly with regard to how its members are selected. Additionally, due to competition with public community health centers, private com*munity health center personnel are not included in the public health committee, resulting in a lack of competition. “With the relatively high workload of social rehabilitation centers, there are four public and one private health service centers in the community. We (the public health committee) have not yet included private health service centers personnel.” (HP-C-5152)*

##### 5.1.1.2 Responsibilities of staff

During the pandemic, the Pingshan District Public Health Committee's policy focus was on containing the spread of the virus, with a particular emphasis on preventing and controlling its spread, such as inspecting individuals considered high risk and coordinating with agencies to promptly address any epidemic situations that may arise within the jurisdiction. Stringent measures were taken to ensure closed-loop management, and publicity efforts were stepped up to enhance epidemic prevention and control policies (22). “*The Public Health Committee's activities centered around pandemic response in 2021.In collaboration with social organizations, enterprises, building administrators, and others, they oversaw vaccination drives and infection prevention and control measures.” (WJJ-C-5151)* In the post-pandemic era, effective measures have been taken to contain the spread of the COVID-19 virus. However, members of the public health committee in Pingshan district continue to adhere to their pandemic-related responsibilities, resulting in a lack of clear guidance for grassroots workers. “*The Public Health Committee may have had more work during the pandemic. Currently but there is not a lot of things to do. And there are hardly any performance requirements. It's basically just about fulfilling assigned tasks.” (SJ-H-5171)* Based on analysis of current policy sources, public health committees in Pingshan District, Shenzhen City, and Guangdong Province have all formulated their construction policies in accordance with the public health committee construction guidelines issued by China's Ministry of Civil Affairs and National Health Commission during the pandemic that showcases a clear top-level design logic. Apart from the pandemic period where workloads were heavier, current workloads are not too heavy. “*The Public Health Committee has scaled down epidemic prevention and control work, as well as the three-person team structure. What remains are patriotic public health work and health knowledge promotion. The Public Health Committee mainly follows its previous work process with minor adaptations.” (SJ-H-5171)*

#### 5.1.2 Work mechanism

##### 5.1.2.1 Model

A work model is the process of an organization adopting methods to achieve specific goals (23). During the pandemic, the primary community in Pingshan District developed a “trinity” work model. Community staff, medical personnel from community health centers, and community police played an important role in key epidemic-related personnel investigation, home quarantine health management, nucleic acid sampling, and vaccine administration. After the establishment of the public health committee, the Pingshan District government included the “three-person team” as members and promoted the organization to extend to various key areas of the community. “*The community plays a significant role at the grassroots level, mainly in the overall coordination of epidemic prevention and control work. Each epidemic prevention and control work, there is cooperation from community police and professional medical personnel from the community health service centers.” (PS-C-5152)”However, in the post-pandemic era, communications among members of the public health committee have decreased. After the end of epidemic prevention and control measures, there has been less interaction between the community and the community health service center. We are also reluctant to trouble them frequently.” (LL-S-5152) “The community health center will not listen to us. The public health committee and the community health center are not subordinate to each other. They mainly rely on personal connections.” (LH-C-5152)* As the public health committees need to assist in providing basic public health services in the community, most of them lack professional members due to medical expertise requirements. Therefore, all leaders of community public health committee recommend that Public Health Bureau can provide them with at least one professional public health staff member. “*At least, the personnel should have basic knowledge of public health, relevant professional backgrounds in public health and professional competences.” (HP-Z-5162)*

##### 5.1.2.2 Work style

Work style refers to the public health committee's efforts in operating in both vertical and horizontal dimensions (24). From the perspective of vertical departmental linkage mechanism, the establishment of public health committees is still within the grassroots administrative system. In terms of the relationship between grassroots administrative organizations and grassroots mass self-governing organizations, it should be a guidance-oriented relationship and an assistance-oriented relationship, rather than a leadership-oriented relationship (25). However, in practical implementation, due to the fact that the work of community public health committee involves professional medical and health services, such as promoting traditional Chinese medicine, mental health services, chronic disease prevention and control, and various forms of health promotion education. The relationship between Public Health departments and community public health committees is not only mentoring and being mentored, but also leadership and being led. Many personnel of community public health committees are part-time employees, which also leads to unsatisfactory results in medical and healthcare services. “*Basically, they only carry out their own activities and indicators within their own jurisdiction, and there is little cooperation with others.” (SH-C-5161) “The sore point of the community public health committee is that currently only the Public Health department is involved. Without a coordination mechanism, the final task still falls on the Public Health department, and the final effect is not ideal. There are two reasons for this. First, the health department does not have the conditions or ability. Second, the social mobilization ability is also not good.” (WJJ-L-5151)*

On the horizontal coordination, public health committee involves public health services such as public health services, patriotic health campaigns, environmental sanitation maintenance, health policy promotion, organizing democratic evaluation of health and health work by residents, etc. Community public health committees need to form collaboration with mass organizations, social organizations, local units, property service enterprises, etc., to play the role of a community public health work network (26). “*Each month, the community health service center has work tasks and indicators that require a certain number of lectures to be completed. Community public health committees organize residents conveniently and hope to serve residents by doing some real work. We have contacted more in terms of giving lectures.” (LH-Y-5152) “We mainly organize activities together with the Party committee and the neighborhood committee. Stock companies, enterprises, and schools will participate in the activities. The community public health committee mainly plays a role in coordination and communication.” (ST-L-5172)*

#### 5.1.3 Job security

##### 5.1.3.1 Site and funding guarantee

Pingshan District has detailed regulations on the construction policy of community public health committees for both office space and funding. The office space of the public health committee is selected to be in the community party group center or the community neighborhood committee. An annual funding of 50,000 yuan is used for renovating the space and purchasing materials. In practical investigation, it was only in 2021 that the community public health committee received funding, but funding has not been implemented in 2022 and 2023. “*In the half of 2021, due to the pandemic, activities couldn't be carried out as usual. We mainly purchased pandemic-related items. The funding was only allocated once. There was no dedicated funding afterward.” (BL-C-5161)* In addition to office expenses, some public health committees used community project budgets to purchase full-time staff from social organizations. However, due to the lack of additional funding, the budget for personnel with medical and health expertise is far below the market average. “*A salary of 93,000 yuan is very low and cannot retain capable individuals. If they can obtain a salary of 160,000 yuan, this treatment is in line with my expectations.” (LH-C-5152)* Reliance on funding has also led to a vicious cycle for community public health committees. As self-governing organizations, community public health committees have no source of funding and can only rely on funding allocated by higher-level departments. However, insufficient funding has resulted in the organization lacking staff with a medical background, thereby exerting a significant negative impact on the entire healthcare system (27).

##### 5.1.3.2 Information system and technical system guarantee

With the continuous development of technologies such as big data, artificial intelligence, the Internet of Things, and cloud computing, telemedicine has been widely applied (28). Shenzhen had also issued relevant policies including “Information and big data management work system,” “Opinions on Comprehensive Promotion of Information Technology in Healthcare Institutions.” These policies had detailed regulations for community medical and health service information systems, then adding modules for family doctor contract services, two-way referrals, and doctor professional information (29). Due to institutional adjustments and cost considerations, the public health committee did not collect data independently and develop an information system. Online communication also enables the transmission of more effective information (30). However, the public health committee also faces difficulties with data latency, data collection, and data exchange. “*We had a specialized information collection team that could collect information. After the institutional adjustment, all the original information collectors were transformed into grid workers. Now they are under the jurisdiction of a wisdom center in Pingshan District. Their job responsibilities have been readjusted, leading to stagnant data and cessation of basic information collection. Most likely all information sources now rely on public security, civil affairs, and hospitals for their data.” (LT-G-5182)”Grid workers do not have permission to retrieve data. They only use their mobile phones to input data. Data retrieval and matching permissions are only available to communities and streets. Additionally, merging and matching data is very difficult and time-consuming. For example, during the pandemic, I once went to the street to retrieve data for two buildings and it took me between half an hour to an hour.” (ZK-Z-5182)* Due to inaccurate and lagging data issues between departments, it is difficult to determine the workload of the public health committee. “*The community sometimes cannot provide accurate data such as older adult, pregnant women, children, etc. There is a high degree of fluidity in our community with people moving in and out every year or switching to other residents*. *It can also be difficult to collect grid data. In addition, even if the community exchanging data with us, the data may not match the data from the District Public Health Department. For example, the District Public Health Department gives us information about a number of older adult of 200, but grid personnel only giving us data with 180 people. The grid personnel's data does not match the District Public Health Department's data. However, higher authorities make us complete workloads based on the larger figure, so the data may not match up.” (LT-H-5181)*

### 5.2 Quantitative findings

#### 5.2.1 Basic information

With a total of 2,000 questionnaires being distributed, we receive 1,990 questionnaires, resulting in a recovery rate of 99.5%. A total of 1,968 valid questionnaires were collected, with a valid response rate of 98.9%. Samples were recovered according to the population proportion of each street and communities. The distribution of gender, age, education level, and other factors among the sample is relatively balanced, and it basically reflects the demographic characteristics of Pingshan District. The descriptive analysis of the respondents is shown in Table 2. Pingshan Subdistrict has the largest population, with 586 interviewees comprising 29.9% of the total sample. Shijing Subdistrict has the smallest population, with only 115 interviewees making up 5.87% of the total sample. The gender distribution is nearly balanced, with roughly equal numbers of males and females. In terms of age, the majority of the interviewed population falls between 19 and 60 years old, accounting for 76.98% of the total sample.

**Table 2:** Basic data statistics.

**Items**	**Categories**	***N***	**Percent (%)**
Jurisdiction of resident's permanent residence	Biling Subdistrict	376	19.18
	Kengzi Subdistrict	139	7.09
	Longtian Subdistrict	440	22.45
	Maluan Subdistrict	304	15.51
	Pingshan Subdistrict	586	29.90
	Shijing Subdistrict	115	5.87
Gender	Female	977	49.64
	Male	991	50.36
Age	18 years old and below	127	6.45
	19–60 years old	1,515	76.98
	Over 61 years old	326	16.57

As demonstrated in Table 3, the satisfaction level with primary public health service capabilities, public health committee awareness, specific responsibilities, and control variables were statistically analyzed from four different perspectives: average values, standard deviation, maximum values, and minimum values. In accordance with the principle that higher values represent higher levels of satisfaction, the average satisfaction level with primary public health service capabilities was 3.935 (with a maximum value of 5), indicating a comparatively high level of satisfaction among residents. In terms of public health committee awareness, the maximum value was 1 (assigned to those who are aware of the establishment). While the minimum value was 0 (assigned to those who are unaware of the establishment). The average public health committee awareness was 0.778, indicating that the majority of residents are aware of the establishment of public health committees. Regarding specific responsibilities, according to the rule that lower values indicate higher degrees of matching, the average demand-supply matching value was 2.750, indicating a mismatch between supply and demand. Information channels, mobilization channels, and emergency response channels were assigned higher values as their capabilities increased. Among the control variables, males were assigned a value of 2 and females were assigned a value of 1. Residents aged 18 and under are assigned a value of 1, residents aged between 19 and 60 are assigned a value of 2, and residents aged 60 and over are assigned a value of 3. In terms of education level, values ranged from 1 to 6, with higher values indicating higher levels of education, ranging from high school, vocational high school, junior college, undergraduate degree, master's degree, to doctoral degree.

**Table 3:** Descriptive statistics for variables.

**Variable names**	**Average values**	**Standard deviation**	**Maximum values**	**Minimum values**
Satisfaction with community health care	3.935	0.880	5.000	1.000
Public health commissioner recognition	0.778	0.415	1.000	0.000
Matching supply to demand	2.750	1.174	5.000	1.000
Information channels	1.798	0.893	5.000	1.000
Mobilization channels	2.631	1.535	8.000	1.000
Emergency response channels	1.832	0.864	5.000	1.000
Males	1.504	0.500	2.000	1.000
Age	2.101	0.469	3.000	1.000
Education level	2.519	1.244	6.000	1.000

#### 5.2.2 Public health committee cognition and satisfaction with grassroots public health service capacity

Table 4 presents the baseline regression results. In Models 1 through 5, the dependent variable is the satisfaction with grassroots public health service capacity, which is an ordinal variable. Therefore, an ordered Probit model is employed. Model 1 introduces the recognition of the public health committee into the baseline model. The results indicate that the regression coefficient of the recognition of the public health committee is significantly positive, suggesting that this recognition has a significant role in enhancing the satisfaction with grassroots public health services. Hypothesis 1 is thus verified. Models 2 to 5 further examine the impact of the four dimensions of public health committee awareness on the satisfaction of grassroots public health services. We sequentially introduce the four core explanatory variables of supply and demand, information channels, organizational mobilization channels, and emergency response channels into the benchmark model. To accurately estimate the impact of awareness on grassroots public health service satisfaction and control for possible regression biases caused by other factors, individual characteristics of respondents are added to each model. By observing the regression results of models 2 to 5, it can be found that the regression coefficient of supply and demand is negative and significant at the 5% level. The regression coefficients of information channels, organizational mobilization channels, and emergency response channels are all positive and significant at the 5% level. Model 6 incorporates all four core explanatory variables and control variables. Similar to the significance of the previous regression equations, supply and demand, organizational mobilization channels, and emergency response channels all show significance. Only the information channel did not show significance. Therefore, Hypothesis 1b is not valid. Model 7 uses OLS regression with grassroots public health service satisfaction as the dependent variable. The variable introduction method in Model 7 is the same as in Model 6. Comparing the estimation results of Model 6 and Model 7, it can be seen that the estimation results of the two models are basically consistent, indicating that the research conclusions of this paper have good robustness.

**Table 4:** Awareness of public health committees and satisfaction with the capacity of primary public health services.

	**Ordered-probit**						**OLS**
	**(1)**	**(2)**	**(3)**	**(4)**	**(5)**	**(6)**	**(7)**
Public health commissioner recognition	0.711^**^ (11.692)						
Matching supply to demand		−0.111^**^ (−5.262)				−0.106^**^ (−4.982)	−0.078^**^ (−4.827)
Information channels			0.220^**^ (7.637)			0.037 (0.960)	0.029 (0.977)
Mobilization channels				0.180^**^ (10.351)		0.145^**^ (7.078)	0.102^**^ (6.703)
Emergency response channels					0.221^**^ (7.490)	0.092^*^ (2.438)	0.059^*^ (2.083)
Males	−0.097^*^ (−1.964)	−0.048 (−0.975)	−0.031 (−0.624)	−0.038 (−0.761)	−0.036 (−0.727)	−0.026 (−0.527)	−0.011 (−0.302)
Age	−0.063 (−1.204)	−0.071 (−1.365)	−0.044 (−0.842)	−0.057 (−1.086)	−0.044 (−0.839)	−0.055 (−1.051)	−0.041 (−0.999)
Education level	0.123^**^ (6.027)	0.166^**^ (8.239)	0.140^**^ (6.892)	0.123^**^ (6.010)	0.220^**^ (7.439)	0.128^**^ (6.192)	0.099^**^ (6.274)
McFadden R Square	0.043	0.020	0.026	0.037	0.026	0.045	0.095
Overall	1,962	1,959	1,962	1,962	1,962	1,959	1,959

^***^, ^**^, ^*^ respectively represent significance levels of 1%, 5%, and 10%.

As for the control variables, age and gender have no significant impact on grassroots public health service satisfaction. The regression coefficient of education level is positive and passes the significance test at the 10% level, indicating that the increase in education level is an important driving force for improving low grassroots public health service satisfaction.

#### 5.2.3 Discussion and testing of possible endogeneity

Table 4 regression results indicate that an increase in public health committee awareness can significantly enhance the satisfaction level of grassroots public health service capabilities. However, there may be a reverse causal relationship between public health committee awareness and satisfaction, where the satisfaction level of public health service capabilities may also influence awareness. This is because the higher the satisfaction level of residents toward public health service capabilities, the more likely they are to actively participate in grassroots public health activities and increase their awareness of public health committee construction. To overcome possible endogeneity biases, we need to find instrumental variables that are related to the explanatory variables but not affected by the satisfaction level of grassroots public health service capabilities. Drawing from existing literature, we select the average education level of the city where the respondent resides as the instrumental variable for supply and demand, organizational mobilization channels, and emergency response channels (31). We use the average education level of individuals in the respondent's neighborhood to construct the instrumental variable for awareness. Existing studies have confirmed that the construction of public health committees, as a mass autonomous organization, relies to some extent on residents' participation in social and economic life under the condition of enhancing their individual potential (32). The average education level of a city affects residents' ability to participate in social construction, and there is no evidence to suggest that the average education level of a city directly affects the satisfaction level of grassroots public health service capabilities.

Table 5 shows that the regression coefficients of public health committee awareness and the three-dimensional variables are still significant at the 5% level, indicating that after considering the endogeneity issues of the model, public health committee awareness and the three-dimensional variables still have a significant positive impact on the satisfaction of grassroots public health service capabilities. This suggests that the conclusions of this study are robust and will not be significantly affected by endogeneity issues.

**Table 5:** Instrumental variable regression results.

	**Model (8)**	**Model (9)**	**Model (10)**	**Model (11)**
Public health commissioner recognition	0.765^**^ (12.783)			
Matching supply to demand		0.098^**^ (−4.682)		
Mobilization channels			0.199^**^ (11.668)	
Emergency response channels				0.244^**^ (8.334)
Control variables	Control	Control	Control	Control
*F*	182.509	125.205	20.910	20.402
Overall	1,959	1,959	1,959	1,959

^***^, ^**^, ^*^ respectively represent significance levels of 1%, 5%, and 10%.

## 6 Discussion

As a grassroots autonomous organization in China, the Public Health Committee should possess autonomy and the capabilities of self-management, self-education, and self-service (33). During the pandemic, in order to fully leverage the protective role of the Public Health Committee at the grassroots level, the government relied on administrative means to lead the establishment of the Public Health Committee, which weakened its autonomy to some extent. But at the grassroots level of epidemic prevention, the role of the public health committee is crucial.

**Figure 2:**
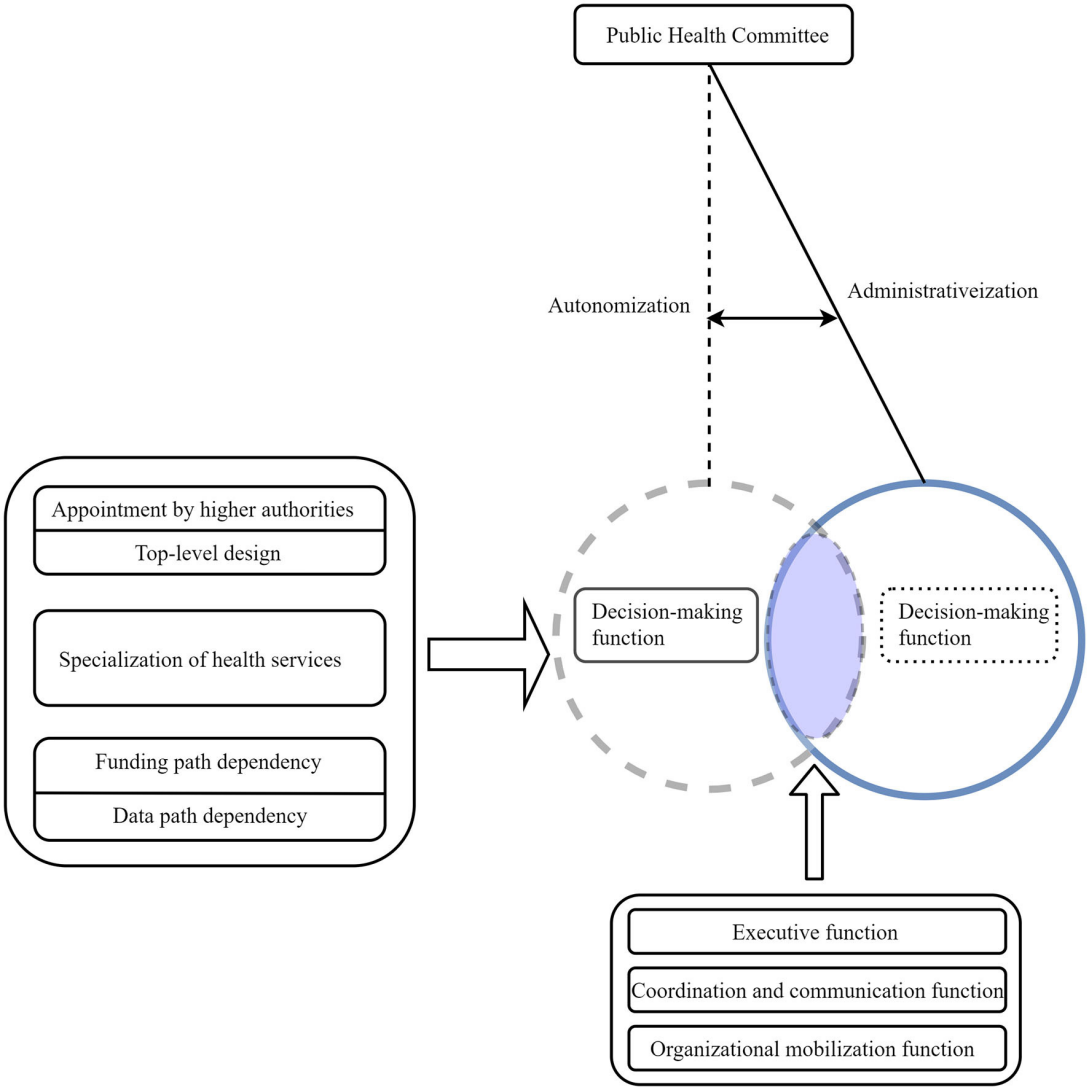
Transformation of the functions of the public health committee.

In the post-epidemic era, this research conducted field research on 23 community public health committees in Pingshan District, Shenzhen City, analyzing their organizational structure, job responsibilities, work security, and capacity building. It was found that the construction of the public health committee still has not completely gotten rid of administrative influence and there is a pendulum effect between “autonomization and administration.” As shown in Figure 2, the construction of the public health committee combines characteristics of both autonomy and administration.

In terms of organizational structure, members of the organization are directly appointed by higher-level departments, and their work responsibilities are carried out in accordance with the requirements of central government documents, with obvious characteristics of top-level design. In terms of work mechanisms, due to the professional nature of public health, all communities lack professional staff. At the same time, due to the professional nature of public health, the public health committee receives professional guidance from higher-level professional departments vertically and cooperates closely with professional community construction service centers horizontally. In terms of work security, the funding sources and data collection of the public health committee have obvious path-dependent characteristics. Due to the sole funding source of the public health committee being government appropriations, there is insufficient office and personnel funding, which in turn leads to a lack of staff with medical backgrounds in the organization. In terms of capacity building, the quantitative analysis in this article found that executive functions, coordination functions, and organizational mobilization functions all have a significant impact on the satisfaction of grassroots public health service capabilities. However, decision-making functions did not have a significant impact on the satisfaction of grassroots public health service capabilities. This is also one of the characteristics of the administratization of autonomous organizations.

As shown in Figure 2, in the early stages of the establishment of public health committees, they were influenced by administrative policies. Such as appointments from higher-level organizations, top-down design characteristics, the specialization of public health and path dependency jointly led to a lack of self-decision-making functions in public health committees. Although public health committees were positioned as mass autonomous organizations that rely on the people for self-management, self-education, and self-service. Their complete separation from the guidance of higher-level departments limited their development. However, the autonomous community organization has completely severed ties with the guidance from higher authorities, which has imposed significant limitations on its development and has also created immense pressure for the residents (34). As a result, autonomous organizations gradually lost their role in grassroots medical and health care and were even gradually forgotten. The establishment of public health committees before the epidemic is a prime example.

In the post-pandemic era, how should public health committees be established? The pendulum model of public health committee establishment can offer us some insights. To prevent excessive autonomy or administration in the establishment of public health committees, public health policies should be continuously refined based on the characteristics of public health committee establishment. During special periods or the early stages of development, the establishment of public health committees should be led by administrative measures. Currently, public health committees in various provinces of China are in the process of being established. The actual establishment of each province is basically following the model of Shenzhen, adopting administrative means and relying on political momentum to promote the development of public health committees. The advantage of administrative measures is that higher-level departments can quickly meet the development needs of public health committees in terms of professionalism, resources, personnel, and material guarantees through the formulation of community medical policies. However, it can also easily lead to a high degree of path dependence in the establishment of public health committees, which is not conducive to the development of public health committees as autonomous organizations. Therefore, in daily establishment or the later stages of establishment, the establishment of public health committees should be led by autonomy, restoring the self-decision-making functions of application. Relying on the masses to self-manage, self-educate, and self-serve the public health committee. For example, absorbing volunteers with professional medical backgrounds into the public health committee to alleviate personnel professional issues. Developing effective professional training programs to alleviate the issue of insufficient personnel expertise (35). Exploring multiple channels for compensatory income by seeking donations from philanthropic enterprises and purchasing third-party services to address issues with office and personnel funding.

Although public health committees have been mentioned in the Constitution of China, it was not until the pandemic that the role of grassroots public health committees attracted attention from society and government departments. The establishment of public health committees is a relatively recent development. Currently, only the public health committees in Beijing, Shanghai, Shenzhen, and Anhui in China are relatively mature. Therefore, there are limitations to the research. Firstly, the research was conducted in Pingshan District, located in Shenzhen, which is a place with a high level of economic development, high level of education among residents, and high level of informatization. Due to differences in economic development levels among various provinces in China, whether the establishment of public health committees has general principles that should being further researched. Secondly, due to considerations of time, cost, and practical situations, this study did not conduct interviews with all members of the public health committee. The collected data can be further expanded to ensure the scientific nature of the research. Finally, as the establishment of public health committees has just begun in China, it has obvious administrative characteristics. The “autonomy-administration” pendulum model was proposed in this paper that needs to be further experienced to see if the autonomy trajectory of public health committee establishment is effective during the mature stage of establishment.

## 7 Conclusion

In China, the construction of public health committees is still in its early stages, with a strong emphasis on administrative policies. As self-governing organizations, the autonomy of committees needs to be further developed. Therefore, to formulate more scientifically-based development policies, it is crucial to consider the pendulum development law of “autonomization-democratization” in light of the current stage of development of public health committees.

## Data availability statement

The raw data supporting the conclusions of this article will be made available by the authors, without undue reservation.

## Ethics statement

The study was approved by the Electronic Information and Artificial Intelligence Ethics Review Committee, Leshan Normal University, Sichuan Province, China (Approval Number 202401121). Ethical review and approval were not required for the current study in accordance with the local legislation and institutional requirements. The participants provided their written informed consent to participate in this study.

## Author contributions

XS: Data curation, Formal analysis, Writing – original draft, Writing – review & editing. XW: Data curation, Formal analysis, Methodology, Writing – original draft, Writing – review & editing.
